# The cross-sectional and prospective associations of parental practices and environmental factors with 24-hour movement behaviours among school-aged Asian children

**DOI:** 10.1186/s12966-024-01574-x

**Published:** 2024-03-04

**Authors:** Natarajan Padmapriya, Anna Fogel, Sarah Yi Xuan Tan, Claire Marie Jie Lin Goh, Shuen Lin Tan, Airu Chia, Anne Hin Yee Chu, Yap Seng Chong, Kok Hian Tan, Shiao-Yng Chan, Fabian Yap, Keith M. Godfrey, Yung Seng Lee, Johan G. Eriksson, Chuen Seng Tan, Jonathan Y. Bernard, Falk Müller-Riemenschneider

**Affiliations:** 1 Saw Swee Hock School of Public Health, National University of Singapore, Singapore, Singapore; 2 Department of Obstetrics & Gynaecology and Human Potential Translational Research Programme, Yong Loo Lin School of Medicine, National University of Singapore, Singapore, Singapore; 3 Singapore Institute for Clinical Sciences (SICS), Agency for Science, Technology and Research (A*STAR), Singapore, Singapore; 4 Department of Paediatrics, Yong Loo Lin School of Medicine, National University of Singapore, Singapore, Singapore; 5 Khoo Teck Puat-National University Children’s Medical Institute, National University Health System, Singapore, Singapore; 6 KK Women’s and Children’s Hospital, Singapore, Singapore; 7 Duke-National University of Singapore, Singapore, Singapore; 8 Lee Kong Chian School of Medicine, Nanyang Technological University, Singapore, Singapore; 9 Medical Research Council Lifecourse Epidemiology Centre, University of Southampton, Southampton, UK; 10 NIHR Southampton Biomedical Research Centre, University of Southampton and University Hospital Southampton NHS Foundation Trust, Southampton, UK; 11 Department of General Practice and Primary Health Care, University of Helsinki and Folkhälsan Research Center, Helsinki, Finland; 12 Université Paris Cité and Université Sorbonne Paris Nord, Inserm, INRAE, Centre for Research in Epidemiology and StatisticS (CRESS), Paris, F-75004 France; 13 Digital Health Center, Berlin Institute of Health, Charité-Universitätsmedizin Berlin, Berlin, Germany

**Keywords:** Movement behaviour, Sleep, Inactivity, Sedentary behaviour, Physical activity, Children, Parental practice, Environment

## Abstract

**Background:**

Parental practices and neighbourhood environmental factors may influence children’s movement behaviours. We aimed to investigate the cross-sectional and prospective associations of parental practices and neighbourhood environmental factors with accelerometer-measured 24-hour movement behaviours (24 h-MBs) among school-aged children in Singapore.

**Methods:**

The Growing Up in Singapore Towards healthy Outcomes (GUSTO) study collected information on dimensions of parental practices and neighbourhood environment at age 5.5 years. Confirmatory factor analyses were performed to generate latent variables and used to compute overall parental practices [involvement in PA + support for PA + control of screen viewing context] and environmental scores [facilities for active play + active mobility facilitators + barriers*-1]. Children wore an accelerometer on their non-dominant wrist for seven consecutive days at ages 5.5 and 8 years. The R-package GGIR 2.6 was used to derive moderate-to-vigorous-intensity physical activity (MVPA), light-intensity physical activity (LPA), inactivity, and total-sleep (napping+night sleep) minutes per day. Associations were determined using compositional data analysis with multivariate linear regression models, taking into account potential confounders.

**Results:**

Among 425 children (48% girls, 59% Chinese), higher parental involvement in PA, parental support for PA and overall parental practices were associated with 24 h-MBs at ages 5.5 and 8 years, specifically with greater time spent in MVPA and less time being inactive relative to the remaining movement behaviours. The corresponding mean changes in the overall 24 h-MB for increasing parental practices from lowest to highest scores (− 2 to + 2 z-scores) indicated potential increases of up to 15-minutes in MVPA, 20-minutes in LPA, 5-minutes in sleep duration, and a reduction of 40-minutes in inactivity at age 5.5 years. At age 8 years, this could translate to approximately 15-minutes more of MVPA, 20-minutes more of LPA, a 20-minute reduction in sleep duration, and a 20-minute reduction in inactivity. Parental control of screen viewing contexts and neighbourhood environmental factors were not associated with 24 h-MBs.

**Conclusions:**

Parental practices but not environmental factors were associated with higher MVPA and lower inactivity among Singaporean children, even at a later age. Further research may provide insights that support development of targeted public health strategies to promote healthier movement behaviours among children.

**Study registration:**

This study was registered on 4th August 2010 and is available online at ClinicalTrials.gov: NCT01174875.

**Supplementary Information:**

The online version contains supplementary material available at 10.1186/s12966-024-01574-x.

## Background

Childhood overweight and obesity are pressing public health issues. The World Health Organization (WHO) estimates that around 41 million children aged 5 years or below are overweight, and nearly half of them reside in Asia [1]. Sufficient time spent in physical activity (PA) and sleep may have favourable effects on preventing obesity and reducing the risk of developing non-communicable diseases (NCDs) at a later age [2–4]. In contrast, sedentary behaviour (SB), which includes screen viewing, may increase the risk of obesity and adverse health outcomes [5, 6]. There is also growing evidence that PA and sleep may have positive impacts on the brain and cognitive function, as well as overall physical, mental, and social health and well-being [7, 8]. PA, SB and sleep behaviours are established in early childhood and track into adulthood [9–11]. Globally, unhealthy behaviours, such as lack of PA, high SB and inadequate sleep among children and adolescents, are emerging threats to public health [12]. The factors that influence the behaviours of children and adolescents are complex and multifaceted, from individual-level factors to various social and environmental factors [13–15]. The Socialization Model for Child Behaviour and socioecological models suggest that parental and neighbourhood environmental factors influence children’s behaviours [16, 17].

Parents play a crucial role in promoting or preventing healthier behaviours in children, especially those aged under 12 years, as children at this age have less volitional control (the process of conscious action) [18, 19]. Previous studies indicate that various parental practices e.g. encouragement, co-participation, role modelling, support for PA, and regulatory support for sleep and screen viewing restriction, may increase PA time, particularly moderate-to-vigorous-intensity PA (MVPA), and/or reduce screen-based SB time and/or promote sufficient sleep among children and adolescents aged 0 to 17 years [20–23]. Such parental practices are likely to vary based on social, cultural, and environmental factors [24].

The neighbourhood environment has been recognized as another important contributor to children’s movement behaviours [16, 17]. Research indicates that children in neighborhoods with recreational facilities and safe infrastructure for walking and cycling tend to be more active and less sedentary [25–27]. Conversely, high crime or traffic areas can limit outdoor activities, leading to more sedentary and poor sleep [28–30]. However, a review of systematic reviews suggests that results of previous studies were less consistent and a large number of studies have not observed associations between environmental factors and PA among children and adolescents aged 1–18 years [26]. More importantly, the vast majority of previous studies have been conducted in Western countries [25, 26], with few studies from Asia to date [14, 31]. Across the world, the neighbourhood environment varies, and it changes rapidly; this may greatly influence the impact of neighbourhood factors on children’s behaviours [32]. Given the complexity of parental factors and neighbourhood environmental factors, it is therefore essential to investigate the region- or country- specific influences of parental and environmental factors on PA, SB and sleep patterns of children.

In addition to the sparsity of investigations from Asia, available evidence on the associations of parental practices and environmental factors on PA, SB and sleep is mainly based on cross-sectional studies and has relied on self/parental-reported behavioural information. Few prospective studies using accelerometer-measured PA, SB and sleep data exist [22, 23, 25, 28]. Moreover, previous research has largely focused on individual behaviours, such as only PA or screen-based SB or sleep [22, 30, 33]. Throughout a 24-hour day, an individual continuously engages in activities of different intensity, i.e. light-intensity PA (LPA), moderate-to-vigorous-intensity PA (MVPA), SB or sleep, and these behaviours are collectively referred to as 24-hour movement behaviour (24 h-MB) [34]. Changes in one of these behaviours inevitably lead to changes in the others, which may impact overall health and well-being [34]. With regards to the impact of parental practices and environmental influences on children’s behaviours, there is little evidence considering the full spectrum of 24 h-MB and accounting for the interdependency of these behaviours. Only one recent study has investigated the associations of environmental facilities for walking with 24 h-MBs, finding that reported walkable neighbourhood was associated with higher MVPA [35]. Considering the existing gaps in the available evidence, we aimed to investigate the cross-sectional and prospective associations of parental practices and neighbourhood environmental factors with accelerometer-measured 24 h-MBs among school-aged Asian children enrolled in a mother-child cohort study.

## Methods

### Study design

The Growing Up in Singapore Towards healthy Outcomes (GUSTO) parent-offspring cohort study recruited pregnant women of Chinese, Malay, or Indian ethnicity who were under 14 weeks of gestation and attending two major public maternity units in Singapore, namely the National University Hospital and KK Women’s and Children’s Hospital. Recruitment took place between June 2009 and October 2010, and the data used in the present study were collected from June 2015 to December 2019. All participants provided written informed consent. The study received ethical approval from the National Healthcare Group Domain Specific Review Board and SingHealth Centralized Institutional Review Board in Singapore (ClinicalTrials.gov: NCT01174875) [36, 37].

### Parental practices and parent perceptions of environmental factors

At child age 5.5 years, parents were asked to complete a questionnaire regarding parental practices and parent perceptions of environmental factors. Parental practices comprised: (i) parental involvement in PA, such as how often they encouraged their child to play outside, how often they were active with their child or in front of him/her, and how often they limited their child’s PA, (ii) parental support for PA, such as how often they took their child to parks, playgrounds, swimming pools, gyms for children, and sports or PA clubs, and whether they enrolled their child in organized sports or other PA programs, and (iii) parental control of screen viewing context, which included how often their child ate meals or snacks while watching television and whether there was a television in his/her bedroom. Neighbourhood environmental factors comprised: (i) the availability of facilities for PA or active play in their local neighbourhood, such as parks, playgrounds, and other open areas, as well as facilities for organized sports and PA, such as swimming pools, gyms, and sports clubs, (ii) facilitators for active mobility or walkability, such as the parental perception of environmental safety, presence of traffic control measures, and access to local shops, and (iii) barriers to active mobility or walkability, such as parental perception of safety concerns regarding traffic, animals, crime, and other potential dangers.

From the self-determination theory perspective, our study assessed several dimensions of parental practices, as well as neighbourhood environmental factors related to movement behaviours. Parental involvement in PA reflects the parental behaviours that can either support or hinder the child’s intrinsic motivation. The dimensions of parental support for PA and neighbourhood factors are aligned with autonomy support within the self-determination theory, as they provide the child with choices and opportunities for engagement in PA. Parental control on screen viewing context relates to the provision of structure within the self-determination theory, as it involves setting boundaries and rules around screen device usage and creating a structured home environment [38–40].

Cronbach’s alpha for internal consistency of parental practices variables, environmental factor variables and all variables in parental practices and environmental factors were 0.82, 0.91 and 0.93, respectively. This suggests relatively high internal consistency among the items measuring parental practices and environmental factors. Confirmatory factor analysis was used to generate latent variables on parental involvement in PA, parental support for PA, parental control on screen viewing context, facilities for active play, facilitators for active mobility and barriers to active mobility. The procedure is detailed in Supplementary Material 1, and the model indices and factor loadings are illustrated in Supplementary Fig. 1. Overall parental practices scores [parental involvement in PA + parental support for PA + parental control on screen viewing context variables], and overall environmental scores [facilities for active play + facilitators for active mobility + (barriers to active mobility* -1)] were computed and used in the analyses.

### Measurement of movement behaviours

ActiGraph GT3X+ (Actigraph Inc., Pensacola, FL) triaxial accelerometers were used to collect movement behaviour data on children at ages 5.5 and 8 years. Accelerometers were initialized with a sampling rate of 80 Hz, and attached to the child’s non-dominant wrist with a non-removable strap during study visits. Parents were instructed to remove the device from the child’s wrist on the ninth day following the visit, allowing for 7 complete days of continuous, 24-hour data capture. Raw data were processed using the GGIR package (version 2.0) in R software [41, 42]. Days with ≥16 hours of activity recordings (from midnight to midnight) were considered valid, and children with at least two valid weekdays and one valid weekend day were included in the analysis. The “2015 van Hees algorithm” was applied to detect sustained inactivity and the night sleep window [43–45]. After visually inspecting the actigraphy data, we determined that sustained inactivity bouts of lasting at least 15-minutes likely represent naps; these bouts were classified as napping time. Total sleep time was calculated (Night sleep + naps). Waking time was classified into three categories: inactivity, LPA, and MVPA, using the Euclidian Norm Minus One (ENMO) values (< 35, 35–200, and ≥ 200 m*g*, respectively, where 1 mg = 0.00981 m.s − ^2^), with prediction equations provided by Hildebrand et al. [46, 47]. While wrist-worn accelerometers do not provide posture information, SB is defined as any waking behavior in a sitting, reclining, or lying down position with energy expenditure ≤1.5 metabolic equivalent tasks (METs) [48, 49]. Therefore, in this study, we used the term “inactivity” as a proxy for SB. Weighted averages of the time spent on each activity across all valid days, with a weighting of 2/5 for weekend days relative to the contribution of weekdays, was used to analyse the data [45]. 

### Covariates

We used a questionnaire to collect information on maternal age and maternal education at recruitment and during the age 5 years visits, respectively. Children’s dates of birth, sex, and ethnicity were retrieved from the hospital’s medical records. At age 5.5 years, the children’s weight (to the nearest gram) and height (to the nearest 0.1 cm) were measured using a weighing scale (SECA model 803) and a stadiometer (SECA model 213, Hamburg, Germany), respectively. These measures were used to calculate body mass index (BMI, kg/m^2^).

### Statistical methods

We evaluated differences between the children included in the study and those excluded using chi-square tests for categorial variables, and Student t-tests for continuous variables. Parental practices, including parental involvement in PA, parental support for PA and parental control on screen viewing context and overall parental practices scores were used as predictor variables. Similarly, environmental scores, including facilities for active play, facilitators for active mobility, barriers to active mobility and overall environmental factors were used as predictor variables.

The compositional data analysis (CoDA) method was used to analyse the accelerometer-measured 24 h-MBs, including MVPA, LPA, inactivity, and sleep duration, as described in the literature [50–52]. The R-package ‘Compositions’ version 2.0–4 was used to execute the CoDA models [53]. The isometric log-ratio (ilr) coordinate sets were constructed using a sequential binary partition, as described by Chastin et al., to express 24 h-MB composition [52]. We constructed four sets of ilr-coordinates for each timepoint. For example, a set of *ilr*-coordinates of MVPA, LPA, inactivity and sleep sequence were: ${ilr}_1=\sqrt{\frac{3}{4}}\mathit{\ln}\left(\frac{MVPA}{\sqrt[3]{LPA\ast Inactivity\ast Sleep}}\right)$; ${ilr}_2=\sqrt{\frac{2}{3}}\mathit{\ln}\left(\frac{LPA}{\sqrt[2]{Inactivity\ast Sleep}}\right)$; ${ilr}_3=\sqrt{\frac{1}{2}}\mathit{\ln}\left(\frac{Inactivity}{Sleep}\right)$. In the CoDA regression models, a set of ilr-coordinates (*ilr*_*1*_, *ilr*_*2,*_ *ilr*_*3*_) were included as an outcome (like in multivariate models). The first ilr-coordinate (*ilr*_*1*_) in each set expressed the proportion of time spent in one behaviour (MVPA or LPA or inactivity or sleep) relative to the remaining three behaviours.

In this study, we employed two approaches to examine the associations of parental practices, neighbourhood environmental factors with children’s movement behaviours. Cross-sectional analysis was conducted at the initial data collection point when children were 5.5 years old. This involved assessing the associations of parental practices and neighbourhood environmental factors (exposure variables) with the accelerometer-measured movement behaviours (outcome variables) at age 5.5 years. For the prospective analysis, we assessed the associations of exposure data collected at age 5.5 years with the outcomes observed at the 8-year visit. We used CoDA multivariate linear regression models to assess the associations of interest, in accordance with the methods described by Dumuid et al. [54]. The models were adjusted for potential confounders including child sex and ethnicity, maternal age, maternal education, and child BMI (kg/m^2^) at age 5.5 years. Estimated marginal means of MVPA, LPA, inactivity and sleep for each unit increase in parental practices and neighbourhood environmental factors (from − 2 to + 2 z-scores) were calculated based on the adjusted model to interpret the results derived from the CoDA regression models. All analyses were conducted in R version 4.1.1 (R Development Core Team, Vienna, Austria), with statistical significance set at *p* < 0.05.

## Results

### Participant characteristics

**Fig. 1:**
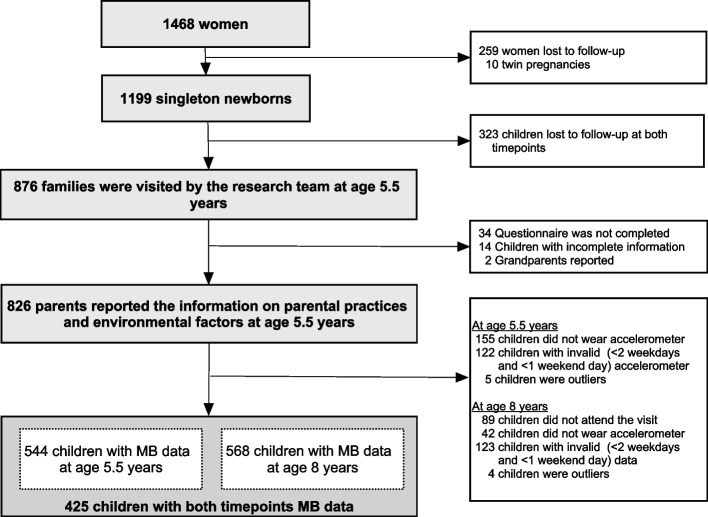
Study flowchart. Footenote: MB, movement behaviour

In the GUSTO cohort study, 826 parents completed the questionnaire on parental practices and environmental factors at ages 5.5 years; among them, 425 children provided valid accelerometer measurements at both ages 5.5 and 8 years (Fig. 1). The characteristics of included children is illustrated in Table 1. An almost equal proportion of girls (48%) and boys (52%) were included in the study, with the majority of children being of Chinese ethnicity (59%). The characteristics of included children did not differ from those of excluded children in terms of sex, ethnicity, maternal age, and BMI (Table 1).

**Table 1:** Comparison of characteristics between children included and excluded from this study in the GUSTO cohort

	Included children (*n* = 425)	Excluded children (*n* = 401)	*p*-value
**Sex, n (%)**			0.860
Girls	204 (48%)	190 (47%)	
Boys	221 (52%)	211 (53%)	
**Ethnicity, n (%)**			0.430
Chinese	249 (59%)	226 (56%)	
Malay	106 (25%)	95 (24%)	
Indian	70 (16%)	80 (20%)	
**Maternal education, n (%)**			**0.028**
Secondary or below	133 (31%)	94 (24%)	
Post-secondary	143 (34%)	136 (34%)	
University	149 (35%)	168 (42%)	
Missing data	0	3	
**Maternal age at recruitment, n (%)**			0.670
< 27 years	93 (22%)	98 (24%)	
27–33 years	173 (41%)	160 (40%)	
> 33 years	159 (37%)	143 (36%)	
**Body Mass Index of children at age 5.5 years** (kg/m^2^), Mean (SD)	15.44 (1.95)	15.52 (2.21)	0.580
**Parental practices at age 5.5 years** (z-score), Mean (SD)			
Parental involvement in PA	−0.04 (0.99)	0.04 (1.01)	0.220
Parental support for PA	−0.07 (0.99)	0.07 (1.01)	**0.044**
Parental control on screen viewing context	0.00 (1.00)	0.00 (1.00)	0.940
Overall parental practices	−0.05 (0.98)	0.05 (1.02)	0.160
**Environmental factors at age 5.5 years** (z-score), Mean (SD)			
Facilities for active play	−0.04 (1.00)	0.04 (1.00)	0.260
Facilitators for active mobility	0.00 (0.97)	0.00 (1.03)	0.900
Barriers to active mobility	0.04 (1.00)	−0.04 (1.00)	0.260
Overall environmental factors	0.00 (0.98)	0.00 (1.02)	0.900
**Accelerometer measured movement behaviours at age 5.5 years **(min/day), Mean (SD)			
Moderate-to-vigorous intensity physical activity	71 (24)	70 (21)	0.620
Light intensity physical activity	344 (49)	343 (49)	0.920
Inactivity (Sedentary behaviour)	488 (69)	482 (65)	0.430
Sleep	537 (42)	542 (47)	0.290
Missing	0	272	
**Accelerometer measured movement behaviours at age 8 years **(min/day), Mean (SD)			
Moderate-to-vigorous intensity physical activity	70 (26)	73 (26)	0.200
Light intensity physical activity	333 (52)	334 (54)	0.810
Inactivity (Sedentary behaviour)	512 (67)	511 (73)	0.970
Sleep	526 (44)	520 (45)	0.230
Missing	0	252	

*SD* standard deviation

*p*-values were determined by Chi-squared test for categorical variables, Student t-test for continuous variables

### Associations of parental practices with 24 h-MBs

Cross-sectional and prospective models showed that parental involvement in PA, parental support for PA and overall parental practices were associated with 24 h-MBs at ages 5.5 and 8 years after accounting for the interdependency of 24 h-MBs and potential confounders (overall *p*-values < 0.05). In contrast, parental control on screen viewing context was not associated with 24 h-MBs at ages 5.5 and 8 years (Table 2). With regards to specific movement behaviours, greater parental involvement in PA, parental support for PA and overall parental practices were associated with a higher amount of time spent in MVPA and a lower amount of time spent in inactivity relative to remaining behaviours. Greater parental involvement in PA, parental support for PA and overall parental practices were associated with shorter total sleep duration relative to remaining behaviours at age 8 years, while parental practices were not associated with total sleep duration at age 5.5 years. No statistically significant associations with LPA were observed.

**Table 2:** Cross-sectional and prospective associations of parental practices with accelerometer-measured 24-hour movement behaviours among children in the GUSTO cohort (*n* = 425)

	Unadjusted model					Adjusted model^a^				
	Relative to remaining behaviours				Overall *p*-value*	Relative to remaining behaviours				Overall *p*-value*
	MVPA	LPA	Inactivity (SB)	Sleep		MVPA	LPA	Inactivity (SB)	Sleep	
	Mean difference (95% CI)	Mean difference (95% CI)	Mean difference (95% CI)	Mean difference (95% CI)		Mean difference (95% CI)	Mean difference (95% CI)	Mean difference (95% CI)	Mean difference (95% CI)	
**24-hour movement behaviours at age 5.5 years**										
Parental involvement	**0.039** **(0.008, 0.071)**	0.013 (0.000, 0.027)	**−0.044** **(− 0.067, − 0.021)**	− 0.008 (− 0.022, 0.005)	**0.002**	**0.039** **(0.007, 0.072)**	0.011 (− 0.003, 0.025)	**− 0.040** **(− 0.064, − 0.016)**	− 0.010 (− 0.025, 0.004)	**0.013**
Parental support for PA	**0.038** **(0.007, 0.070)**	0.009 (− 0.005, 0.023)	**− 0.042** **(− 0.065, − 0.019)**	−0.006 (− 0.019, 0.008)	**0.003**	**0.042** **(0.009, 0.074)**	0.005 (− 0.009, 0.020)	**− 0.039** **(− 0.063, − 0.015)**	−0.008 (− 0.023, 0.007)	**0.018**
Parental control on screen viewing context	−0.007 (− 0.039, 0.024)	0.003 (− 0.011, 0.016)	−0.008 (− 0.031, 0.014)	0.013 (0.000, 0.027)	0.085	0.004 (− 0.029, 0.036)	−0.005 (− 0.019, 0.009)	−0.010 (− 0.034, 0.014)	0.011 (− 0.003, 0.026)	0.105
Overall parental practices	0.030 (− 0.002, 0.062)	0.011 (− 0.003, 0.025)	**−0.041** **(− 0.064, − 0.018)**	0.000 (− 0.014, 0.014)	**0.002**	**0.037** **(0.004, 0.071)**	0.005 (− 0.010, 0.019)	**−0.039** **(− 0.064, − 0.014)**	−0.003 (− 0.018, 0.012)	**0.012**
**24-hour movement behaviours at age 8 years**										
Parental involvement	**0.048** **(0.015, 0.082)**	0.015 (−0.001, 0.031)	**−0.036** **(− 0.058, − 0.013)**	**−0.028** **(− 0.043, − 0.012)**	**0.001**	**0.043** **(0.010, 0.076)**	0.007 (− 0.009, 0.023)	−0.023 (− 0.046, 0.000)	**−0.027** **(− 0.043, − 0.011)**	**0.009**
Parental support for PA	**0.040** **(0.007, 0.073)**	**0.017** **(0.002, 0.033)**	**−0.034** **(− 0.057, − 0.012)**	**−0.023** **(− 0.038, − 0.008)**	**0.003**	**0.037** **(0.003, 0.070)**	0.008 (− 0.008, 0.024)	−0.022 (− 0.045, 0.002)	**−0.023** **(− 0.039, − 0.007)**	**0.038**
Parental control on screen viewing context	0.000 (− 0.033, 0.033)	**0.015** **(0.000, 0.031)**	−0.015 (− 0.037, 0.008)	−0.001 (− 0.016, 0.014)	0.190	0.012 (− 0.021, 0.045)	0.009 (− 0.007, 0.025)	−0.014 (− 0.037, 0.010)	−0.007 (− 0.023, 0.009)	0.546
Overall parental practices	**0.038** **(0.004, 0.072)**	0.021 (0.005, 0.037)	**−0.037** **(− 0.060, − 0.014)**	**−0.022** **(− 0.038, − 0.007)**	**0.001**	**0.040** **(0.006, 0.074)**	0.011 (− 0.006, 0.027)	**−0.026** **(− 0.049, − 0.002)**	**−0.025** **(− 0.041, − 0.009)**	**0.016**

*CI* confidence interval, *SB* sedentary behaviour, *LPA* Light physical activity, *MVPA* Moderate-to-vigorous physical activity

^a^Models were adjusted for sex, ethnicity, BMI at age 5.5 years and maternal age and education

Results are based on a compositional data analysis, multivariate linear regression models

*Type II MANOVA Tests: Pillai test statistics

**Fig. 2:**
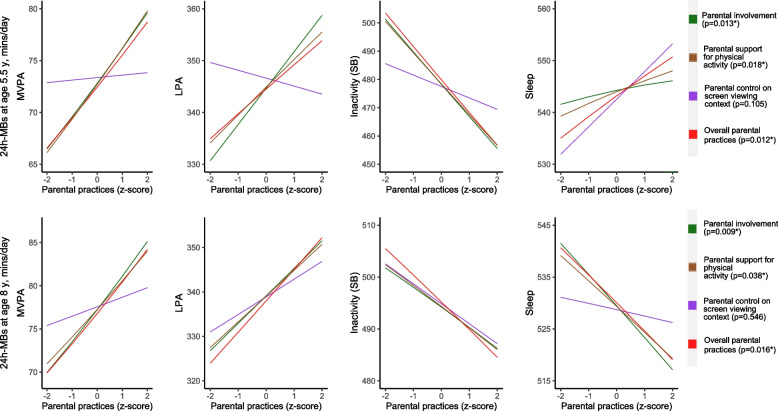
Estimated means of children's 24-hour movement behaviours at ages 5.5 and 8 years for each unit change in parental practices at age 5.5 years in the GUSTO cohort (*n* = 425). Footnote: MB, movement behaviour; MVPA, Moderate-to-vigorous intensity physical activity; LPA, Light physical activity; SB, sedentary behaviour; y, year; mins/d, minutes per day. Models were adjusted for sex, ethnicity, BMI at age 5.5 years and maternal age and education. Results are based on a compositional data analysis, multivariate linear regression (adjusted) models; *Type II MANOVA Tests: Pillai test statistics

The estimated changes in movement behaviours resulting from changes in parental practices are illustrated in Fig. 2. The estimated means show that increasing parental involvement in PA, parental support for PA, and overall parental practices from a z-score of − 2 to one of + 2 could result in an up to 15-minutes increase in MVPA, 20-minutes increase in LPA, 5 minutes increase in sleep duration, and a reduction in inactivity by up to 40-minutes per day at age 5.5 years. Similarly, at age 8 years, the same changes in parental practices could translate to an approximately 15-minutes increase in MVPA, 20-minutes increase in LPA, a 20-minute reduction in sleep, and a 20-minute reduction in inactivity duration per day.

### Associations of environmental factors with 24 h-MBs

Neighbourhood facilities for active play, facilitators and barriers to active mobility and overall environmental factors were not associated with 24 h-MB at ages 5.5 and 8 years after accounting for the interdependency of 24 h-MBs and potential confounders (overall *p*-values > 0.05) (Table 3). With regards to specific movement behaviours, unadjusted models showed that more facilities for active play were associated with a higher amount of time spent in LPA and a lower amount of time in inactivity, relative to the remaining behaviours at age 8 years. Similarly, a higher score of overall environmental factors were associated with a lower amount of time spent in inactivity relative to the remaining behaviours at age 8 years in the unadjusted model. However, these associations were no longer statistically significant after adjusting for confounders.

**Table 3:** Cross-sectional and prospective associations of environmental factors with accelerometer-measured 24-hour movement behaviours among children in the GUSTO cohort (*n* = 425)

	Unadjusted model					Adjusted model^a^				
	Relative to remaining behaviours				Overall *p*-value*	Relative to remaining behaviours				Overall *p*-value*
	MVPA	LPA	Inactivity (SB)	Sleep		MVPA	LPA	Inactivity (SB)	Sleep	
	Mean difference (95% CI)	Mean difference (95% CI)	Mean difference (95% CI)	Mean difference (95% CI)		Mean difference (95% CI)	Mean difference (95% CI)	Mean difference (95% CI)	Mean difference (95% CI)	
**24-hour movement behaviours at age 5.5 years**										
Facilities for active play	0.018 (−0.013, 0.049)	0.007 (− 0.006, 0.021)	**−0.026** **(− 0.049, − 0.004)**	0.001 (− 0.013, 0.015)	0.072	0.020 (− 0.012, 0.051)	0.003 (− 0.011, 0.017)	−0.023 (− 0.047, 0.001)	0.000 (− 0.015, 0.014)	0.224
Facilitators for active mobility	0.004 (− 0.028, 0.036)	0.000 (− 0.014, 0.014)	−0.009 (− 0.032, 0.015)	0.005 (− 0.009, 0.019)	0.222	−0.003 (− 0.035, 0.029)	0.000 (− 0.014, 0.014)	−0.003 (− 0.027, 0.021)	0.006 (− 0.008, 0.021)	0.357
Barriers to active mobility	− 0.006 (− 0.038, 0.025)	0.002 (− 0.012, 0.016)	−0.007 (− 0.030, 0.016)	0.011 (− 0.003, 0.025)	0.596	−0.011 (− 0.043, 0.020)	0.000 (− 0.014, 0.013)	0.000 (− 0.024, 0.024)	0.012 (− 0.003, 0.026)	0.741
Overall environmental factors	0.006 (− 0.026, 0.038)	0.004 (− 0.010, 0.018)	−0.017 (− 0.041, 0.006)	0.008 (− 0.006, 0.022)	0.121	0.001 (− 0.031, 0.034)	0.001 (− 0.013, 0.015)	−0.010 (− 0.035, 0.014)	0.008 (− 0.006, 0.023)	0.323
**24-hour movement behaviours at age 8 years**										
Facilities for active play	0.025 (−0.008, 0.058)	**0.019** **(0.003, 0.034)**	**−0.031** **(− 0.053, − 0.008)**	−0.013 (− 0.029, 0.002)	**0.014**	0.022 (− 0.010, 0.054)	0.010 (− 0.005, 0.026)	−0.020 (− 0.043, 0.002)	−0.012 (− 0.027, 0.003)	0.222
Facilitators for active mobility	0.025 (−0.009, 0.059)	0.006 (− 0.010, 0.022)	−0.020 (− 0.043, 0.003)	−0.011 (− 0.026, 0.005)	0.282	0.012 (− 0.021, 0.045)	0.004 (− 0.012, 0.019)	−0.010 (− 0.033, 0.013)	−0.006 (− 0.021, 0.010)	0.772
Barriers to active mobility	0.027 (−0.006, 0.060)	0.004 (− 0.011, 0.020)	−0.022 (− 0.045, 0.000)	−0.009 (− 0.024, 0.007)	0.344	0.017 (− 0.015, 0.049)	0.000 (− 0.015, 0.015)	−0.011 (− 0.034, 0.012)	−0.006 (− 0.021, 0.009)	0.830
Overall environmental factors	0.033 (0.000, 0.067)	0.012 (−0.004, 0.028)	**−0.031** **(− 0.054, − 0.008)**	−0.014 (− 0.030, 0.002)	**0.044**	0.022 (− 0.011, 0.055)	0.006 (− 0.010, 0.022)	−0.018 (− 0.041 0.005)	−0.010 (− 0.026 0.005)	0.434

*CI* confidence interval, *SB* sedentary behaviour, *LPA* Light physical activity, *MVPA* Moderate-to-vigorous physical activity

^a^Models were adjusted for sex, ethnicity, BMI at age 5.5 years and maternal age and education

Results are based on a compositional data analysis, multivariate linear regression models

*Type II MANOVA Tests: Pillai test statistics

### Sensitivity analysis

The results of sensitivity analysis with maximum sample sizes at age 5.5 (*n* = 544) and age 8 (*n* = 568) years are presented in Supplementary Tables 1 and 2. The analyses confirm or even strengthen the aforementioned results for parental practices. Results for environmental factors indicated that more neighbourhood facilities for active play were significantly associated with a higher amount of time spent in MVPA relative to remaining behaviours at age 5.5 years after accounting for the interdependency of 24 h-MBs and potential confounders. More neighbourhood facilities for active play and higher overall environmental scores were associated with a lower amount of time spent in inactivity at age 8 years after accounting for the interdependency of 24 h-MBs and potential confounders.

## Discussion

The GUSTO study is the first prospective multi-ethnic cohort study to investigate the associations of parental practices and environmental factors with the full spectrum of 24 h-MB among Asian children. We used an integrated approach to study time-use, thereby accounting for the interdependence of different movement behaviours. Our study demonstrates that parental practices were consistently associated with 24 h-MBs among school-aged children. In particular, higher levels of parental involvement in PA and parental support for PA were associated with a higher amount of time spent in MVPA and a lower amount of time spent in inactivity. These associations translated into improvements in MVPA and LPA, as well as corresponding reductions in inactivity within the context of 24 h-MB. On the other hand, no consistent associations between neighbourhood environmental factors and children’s 24 h-MBs were observed.

Given the variability in how parental practices were assessed and analysed in previous studies [18], it is important to consider the specific dimensions of parental practices being examined. The literature suggests that parental role modelling and co-participation are strongly associated with children’s PA and/or SB [20, 22, 55]. Reviews of previous studies suggest that children and adolescents (aged 0–18 years) whose parents encouraged or supported them to engage in PA or organized sports may have higher levels of PA or higher amount of time in PA, particularly MVPA, and/or lower SB [13, 18, 22, 55]. However, a systematic review of the associations of parental encouragement or support with outdoor play showed mixed results, with either positive or no associations with outdoor play among children aged 0–12 years [23]. The associations of parental involvement in PA and parental support for PA with LPA were unclear, and there is no research available on the associations with sleep [55]. Moreover, previous studies have not accounted for the interdependency between different movement behaviours. The present study is unique in that it examines the associations between parental practices and 24 h-MBs in children while accounting for their interdependency. Our study shows that parental practices were associated with the full spectrum of 24 h-MBs cross-sectionally and prospectively. Greater parental practices were associated with a higher amount of time spent in MVPA and a lower amount of time spent in inactivity relative to the remaining movement behaviours, with the associations driven by parental involvement in PA and parental support on PA. We observed that more involved and supportive parental practices were associated with a higher amount of time spent in LPA, though the associations did not reach statistical significance. Using a prospective study design, accelerometers to measure 24 h-MBs objectively, and the CoDA approach consistent with the Framework for Viable Integrative Research in Time-Use Epidemiology (VIRTUE) [34], our findings strengthen the existing evidence considerably. At the same time our findings are broadly consistent with the results of previous research on PA and/or SB that has shown that parental encouragement, role modelling, co-participation, and support for PA are associated with higher levels of PA and/or lower levels of SB in children and adolescents [13, 18, 20, 22, 55]. Possible explanations for these findings include the following: Firstly, parental involvement in the form of encouragement, role modelling, and co-participation in PA, creates a social environment that promotes active lifestyles within the family. When parents actively engage in PA themselves and provide support and encouragement to their children, it establishes a normative behaviour and reinforces the value of being physically active. Secondly, by supporting children’s PA in the form of improved access to neighbourhood facilities and endorsing or enrolling into organized sports, parents create opportunities for their children to engage in MVPA but also limit inactivity [38–40].

Analyses of the estimated time spent in each movement behaviour showed that increased parental involvement in PA and parental support for PA could lead to an increase in MVPA and LPA by up to 15- and 20-minutes per day, respectively, and corresponding reduction in inactivity by up to 40 minutes per day. These findings are noteworthy as the potential increase in PA accounts for a substantial portion of the recommended daily amounts of MVPA (60 minutes of MVPA per day) and estimated changes would result in a significant increase in the proportion of children meeting PA recommendations [56, 57]. Moreover, an increase in LPA might have a significant impact on health outcomes. Emerging research, including studies by Agbaje et al. and Segura-Jiménez et al., has begun to shed light on this aspect. These studies suggest that higher levels of LPA are associated with favourable health outcomes, such as lower fat mass, lower cholesterol, and reduced inflammatory effects in children and adolescents [58–61]. This evidence reinforces the importance of acknowledging the influence of parental behaviour on children’s engagement in LPA in the paediatric population. These findings further highlight the critical role of parental practices in shaping children’s movement behaviours and emphasize the significance of parental involvement in PA and support in promoting an active lifestyle from a young age. However, experimental studies are necessary to ascertain whether actual improvements in parental involvement, parental support, and overall parental practices would lead to meaningful changes in 24 h-MB.

Our study showed inconsistent findings for the associations between parental practices and children’s sleep duration. For instance, we did not observe any significant cross-sectional associations between parental practices and sleep duration, while parental practices, including higher parental involvement in PA and support for PA, were associated with shorter sleep duration prospectively. Interpretation of these findings is not straightforward. On the one hand, parental practices for PA do not target children’s sleep practices directly, which may explain inconsistent findings. Unfortunately, our study did not collect information on sleep-related parental practices, which could be an important consideration for future research. On the other hand, greater supportiveness for PA may come at the expense of deliberately or accidentally reducing children’s sleep duration. One possible explanation for the observed differences could be that as children grow, there is a natural reduction in their sleep duration. In the Singapore context, at age 5.5 years, children were in kindergarten where children may have napping time, while at age 8 years, they were in primary school where napping time was not possible. This could mean that at age 5.5 years, parental practices that encouraged PA may have led to less time spent in inactivity, but at age 8 years, PA was increased at the expense of both inactivity and napping/total sleeping time. This raises the importance of considering the significance of adequate sleep for children’s overall well-being and for parental practices to adopt a 24-hour paradigm that considers PA, SB, and sleep collectively.

In the present study, parental control over screen viewing context, which includes restrictions on eating meals or snacks while watching TV and avoiding televisions in the bedroom, did not show significant associations with 24 h-MBs both cross-sectionally and prospectively. It is noteworthy that previous studies did not consider the interdependency of 24 h-MBs, making it challenging to draw direct comparisons between the results of different studies. Nevertheless, literature reviews suggest that eating meals while watching TV or having screen devices in a child’s bedroom may be associated with higher screen-based SB time and/or shorter sleep duration [22, 55, 62]. While our study examined parental control over screen viewing context, we did not consider rules on the amount of screen viewing time. This distinction is important because rules on the quantity of screen viewing could have a more direct impact on children’s screen-based SB or total SB [63]. Furthermore, in the context of Singaporean school children who spend significant time in school and in extracurricular tuition [64], screen viewing may not reflect total SB well. Instead, other sedentary activities, such as sitting during classes or doing homework, may contribute substantially to overall SB, which could explain the lack of associations with parental practices targeting screen viewing contexts. Future research should encompass a wider range of parental practices on SB, including rules on recreational screen viewing duration and academic activities, to capture a more comprehensive understanding of parental influence on children’s 24 h-MBs.

A recent literature review has suggested that various environmental factors, such as recreational facilities, open spaces, lighting, traffic safety, and access to destinations, are positively associated with total PA and/or transportation-related PA in children and adolescents aged 1–18 years [26]. However, the authors noted that these positive associations were mostly based on self-reported data and that recreational neighbourhood facilities were not associated with device-measured total PA among children [26]. In terms of sleep, a systematic review found that lower neighbourhood safety was associated with shorter sleep duration and lower sleep quality [30]. Another systematic review suggested that very few studies have reported on the associations between neighbourhood facilities and SB and the results were inconclusive [33]. A study among Canadian children aged 8–10 years appears to be the only one that investigated the associations of neighbourhood walkability with accelerometer measured 24 h-MBs after accounting for the independency of movement behaviours [35]. The authors reported that more walkable neighbourhoods were associated with higher MVPA, and observed decreases in LPA, SB and sleep relative to MVPA [35]. The present study revealed that parental support for using environmental facilities for PA was associated with higher MVPA and lower inactivity among children. However, no associations between environmental facilities for PA and 24 h-MB were observed in the main analyses. Interestingly, our sensitivity analysis with a larger sample size showed that a higher number of neighbourhood facilities seemed to be linked to more time spent in MVPA and less time spent in inactivity, although these associations were not consistently observed across all models. It is noteworthy that Singapore, where the study was conducted, received the highest grade (A+) for community and environmental indicators of PA in the Global Matrix 4.0 [65], indicating that more than 90% of children have access to neighbourhoods supportive of PA [66]. This illustrates that Singapore has made significant efforts to provide accessible and supportive environments for PA. Similarly, we did not find any associations of facilitators and barriers to active mobility with 24 h-MBs among Singaporean children. It is conceivable that in a densely populated and relatively homogeneous high income country like Singapore, where the majority has access to high-quality recreational spaces and infrastructure, the associations between neighbourhood environmental factors and 24 h-MBs may be more nuanced [67]. Future research in Singapore may therefore require more refined and objective measures of the built and natural environments to address these questions more adequately.

Strengths of our study include the prospective design, use of multiple timepoint accelerometer-measured data, and an integrated time-use data analysis approach to investigate the associations of parental practices and neighbourhood environmental factors with 24 h-MBs. Limitations include that our study is not fully representative of the entire Singaporean population, and only about 40% of the original study population completed both time points, which could affect the generalizability and statistical power of our findings. We used wrist-worn accelerometers to measure movement behaviour, which was associated with high compliance, but did not measure posture-based SB. Although inactivity time can be a proxy for SB and we used the SB cut-point derived in a laboratory study; potential misclassification of SB time and napping time cannot be disregarded [41, 42, 47]. This limitation necessitates cautious interpretation of our results, particularly regarding the inconsistent findings related to sleep duration. Unmeasured residual confounding factors, such as factors related to schools and peers, sleep-related parental practices, changes in parental practices and environmental factors between ages 5.5 and 8 years, and cardiometabolic confounders, cannot be discounted. Notably, the prospective analysis did not account for changes in the parental practices and neighbourhood environmental factors and 24 h-MBs over time, as the parental practices and neighbourhood environmental factors data was not available at age 8 years. In addition, the lack of detailed objectively measured environmental data is a limitation of this study. Nonetheless, our findings are important in taking a contemporary perspective to investigate predictors of the full spectrum of 24 h-MBs. This information has the potential to shed light on research possibilities and aid in the development of strategies to enhance movement behaviours for better health and well-being.

## Conclusions

This study used an integrated time-use approach to provide new insights into the associations of parental practices and neighbourhood environmental factors with the full spectrum of 24 h-MBs. Our study illustrates the important influence of parental practices on movement behaviours among Asian school-aged children in Singapore. Greater parental involvement in PA, parental support for PA and overall parental practices were associated with more time spent in MVPA and less time spent in inactivity, potentially translating into meaningful improvements in PA and reduction in inactivity. Associations with sleep duration were inconsistent but indicate that parental practices may benefit from considering PA, SB, and sleep, avoiding promotion of PA at the expense of sleep. On the other hand, in the densely populated high income city-state of Singapore with access to relatively high-quality recreational spaces, we observed limited associations between neighbourhood environmental factors and 24 h-MBs. Further research using more granular and objective approaches to investigate the built and natural environments is warranted to better understand the relationships between details of neighbourhood environmental facilities and 24 h-MBs. Ultimately, this research may contribute to the development of strategies targeting movement behaviours holistically and promoting health and well-being of children in Singapore and more widely.

### Supplementary Information

**Supplementary Material 1.**

**Supplementary Material 2.**

**Supplementary Material 3.**

## Data Availability

The dataset supporting the conclusions of this article can be made available upon request and after approval by the GUSTO Executive Committee.

## References

[1] World Health Organization. Obesity and overweight. https://www.who.int/news-room/fact-sheets/detail/obesity-and-overweight. Accessed June 2023.

[2] World Health Organization. Childhood overweight and obesity. In: Global Strategy on Diet, Physical Activity and Health. 2016. https://www.who.int/news-room/questions-and-answers/item/noncommunicable-diseases-childhood-overweight-and-obesity . Accessed June 2023

[3] Reilly JJ, Hughes AR, Gillespie J, Malden S, Martin A (2019). Physical activity interventions in early life aimed at reducing later risk of obesity and related non-communicable diseases: a rapid review of systematic reviews. Obes Rev.

[4] Ruan H, Xun P, Cai W, He K, Tang Q (2015). Habitual sleep duration and risk of childhood obesity: systematic review and dose-response Meta-analysis of prospective cohort studies. Sci Rep.

[5] Tremblay MS, LeBlanc AG, Kho ME, Saunders TJ, Larouche R, Colley RC (2011). Systematic review of sedentary behaviour and health indicators in school-aged children and youth. Int J Behav Nutr Phys Act.

[6] Pinto AJ, Bergouignan A, Dempsey PC, Roschel H, Owen N, Gualano B (2023). Physiology of sedentary behavior. Physiol Rev.

[7] Rollo S, Antsygina O, Tremblay MS (2020). The whole day matters: understanding 24-hour movement guideline adherence and relationships with health indicators across the lifespan. J Sport Health Sci.

[8] Alanazi YA, Sousa-Sá E, Chong KH, Parrish AM, Okely AD. Systematic review of the relationships between 24-hour movement Behaviours and health indicators in school-aged children from Arab-speaking countries. Int J Environ Res Public Health. 2021;18(16). 10.3390/ijerph18168640 PMC8391650 34444388

[9] Craigie AM, Lake AA, Kelly SA, Adamson AJ, Mathers JC (2011). Tracking of obesity-related behaviours from childhood to adulthood: a systematic review. Maturitas..

[10] Biddle SJ, Pearson N, Ross GM, Braithwaite R (2010). Tracking of sedentary behaviours of young people: a systematic review. Prev Med.

[11] Sivertsen B, Harvey AG, Pallesen S, Hysing M (2017). Trajectories of sleep problems from childhood to adolescence: a population-based longitudinal study from Norway. J Sleep Res.

[12] Tapia-Serrano MA, Sevil-Serrano J, Sánchez-Miguel PA, López-Gil JF, Tremblay MS, García-Hermoso A (2022). Prevalence of meeting 24-hour movement guidelines from pre-school to adolescence: a systematic review and meta-analysis including 387,437 participants and 23 countries. J Sport Health Sci.

[13] Biddle SJH, Atkin AJ, Cavill N, Foster C (2011). Correlates of physical activity in youth: a review of quantitative systematic reviews. Int Rev Sport Exerc Psychol.

[14] Chen B, Kui KY, Padmapriya N, Müller AM, Müller-Riemenschneider F (2022). Correlates of sedentary behavior in Asian preschool-aged children: a systematic review. Obes Rev.

[15] Zhang Z, Sousa-Sá E, Pereira JR, Okely AD, Feng X, Santos R (2021). Correlates of sleep duration in early childhood: a systematic review. Behav Sleep Med.

[16] Lee EY, Hesketh KD, Rhodes RE, Rinaldi CM, Spence JC, Carson V (2018). Role of parental and environmental characteristics in toddlers' physical activity and screen time: Bayesian analysis of structural equation models. Int J Behav Nutr Phys Act.

[17] Carson V, Hesketh KD, Rhodes RE, Rinaldi C, Rodgers W, Spence JC (2017). Psychometric properties of a parental questionnaire for assessing correlates of Toddlers' physical activity and sedentary behavior. Meas Phys Educ Exerc Sci.

[18] Beets MW, Cardinal BJ, Alderman BL (2010). Parental social support and the physical activity-related behaviors of youth: a review. Health Educ Behav : Off Publ Soc Public Health Educ..

[19] Pyper E, Harrington D, Manson H (2016). The impact of different types of parental support behaviours on child physical activity, healthy eating, and screen time: a cross-sectional study. BMC Public Health.

[20] Keyes BL, Wilson KS (2021). Influence of parental physical activity and sedentary behavior on young children: considering time together. Res Q Exerc Sport.

[21] Goncalves WSF, Byrne R, de Lira PIC, Viana MT, Trost SG (2022). Adherence to 24-hour movement guidelines among rural Brazalian preschool children: associations with parenting practices. Int J Behav Nutr Phys Act.

[22] Xu H, Wen LM, Rissel C (2015). Associations of parental influences with physical activity and screen time among young children: a systematic review. J Obes.

[23] Boxberger K, Reimers AK. Parental correlates of outdoor play in boys and girls aged 0 to 12-a systematic review. Int J Environ Res Public Health. 2019;16(2). 10.3390/ijerph16020190 PMC6351982 30641874

[24] Lin G-X, Mikolajczak M, Keller H, Akgun E, Arikan G, Aunola K (2023). Parenting culture(s): ideal-parent beliefs across 37 countries. J Cross-Cult Psychol.

[25] Pont K, Ziviani J, Wadley D, Bennett S, Abbott R (2009). Environmental correlates of children's active transportation: a systematic literature review. Health Place..

[26] Prince SA, Lancione S, Lang JJ, Amankwah N, de Groh M, Jaramillo Garcia A (2022). Examining the state, quality and strength of the evidence in the research on built environments and physical activity among children and youth: An overview of reviews from high income countries. Health Place.

[27] An R, Shen J, Yang Q, Yang Y (2019). Impact of built environment on physical activity and obesity among children and adolescents in China: a narrative systematic review. J Sport Health Sci.

[28] Gerards SMPL, Van Kann DHH, Kremers SPJ, Jansen MWJ, Gubbels JS (2021). Do parenting practices moderate the association between the physical neighbourhood environment and changes in children’s time spent at various physical activity levels? An exploratory longitudinal study. BMC Public Health.

[29] do Carmo AS, Rodrigues D, Nogueira H, Mendes LL, dos Santos LC, Gama A (2020). Influence of parental perceived environment on physical activity, TV viewing, active play and body mass index among Portuguese children: a mediation analysis. Am J Hum Biol.

[30] Mayne SL, Mitchell JA, Virudachalam S, Fiks AG, Williamson AA (2021). Neighborhood environments and sleep among children and adolescents: a systematic review. Sleep Med Rev.

[31] Lu C, Shen T, Huang G, Corpeleijn E (2022). Environmental correlates of sedentary behaviors and physical activity in Chinese preschool children: a cross-sectional study. J Sport Health Sci.

[32] Ding D, Adams MA, Sallis JF, Norman GJ, Hovell MF, Chambers CD (2013). Perceived neighborhood environment and physical activity in 11 countries: do associations differ by country?. Int J Behav Nutr Phys Act.

[33] Stierlin AS, De Lepeleere S, Cardon G, Dargent-Molina P, Hoffmann B, Murphy MH (2015). A systematic review of determinants of sedentary behaviour in youth: a DEDIPAC-study. Int J Behav Nutr Phys Act.

[34] Pedisic Z, Dumuid D, Olds TS (2017). Integrating sleep, sedentary behaviour, and physical activity research in the emerging field of time-use epidemiology: definitions, concepts, statistical methods, theoretical framework, and future directions. Kinesiology (Zagreb, Croatia).

[35] Bird M, Datta GD, Chinerman D, Kakinami L, Mathieu M-E, Henderson M (2022). Associations of neighborhood walkability with moderate to vigorous physical activity: an application of compositional data analysis comparing compositional and non-compositional approaches. Int J Behav Nutr Phys Act.

[36] Soh SE, Chong YS, Kwek K, Saw SM, Meaney MJ, Gluckman PD (2014). Insights from the growing up in Singapore towards healthy outcomes (GUSTO) cohort study. Ann Nutr Metab.

[37] Soh SE, Tint MT, Gluckman PD, Godfrey KM, Rifkin-Graboi A, Chan YH (2014). Cohort profile: growing up in Singapore towards healthy outcomes (GUSTO) birth cohort study. Int J Epidemiol.

[38] Roberta Di P, Andrea R, Ignacio Jáuregui L (2018). Parenting influences on child obesity-related behaviors: a self-determination theory perspective. Weight Loss.

[39] Ha AS, Ng JYY, Lonsdale C, Lubans DR, Ng FF (2019). Promoting physical activity in children through family-based intervention: protocol of the “active 1 + FUN” randomized controlled trial. BMC Public Health.

[40] Teixeira PJ, Carraça EV, Markland D, Silva MN, Ryan RM (2012). Exercise, physical activity, and self-determination theory: a systematic review. Int J Behav Nutr Phys Act.

[41] van Hees VT, Gorzelniak L, Dean León EC, Eder M, Pias M, Taherian S (2013). Separating movement and gravity components in an acceleration signal and implications for the assessment of human daily physical activity. PLoS One.

[42] Jairo HM, Alex VR, Florian H, Séverine S, TvH V (2019). GGIR: A Research Community–Driven Open Source R Package for Generating Physical Activity and Sleep Outcomes From Multi-Day Raw Accelerometer Data. J Meas Phys Behav..

[43] van Hees VT, Sabia S, Anderson KN, Denton SJ, Oliver J, Catt M (2015). A novel, open access method to assess sleep duration using a wrist-worn accelerometer. PLoS One.

[44] van Hees VT, Sabia S, Jones SE, Wood AR, Anderson KN, Kivimäki M (2018). Estimating sleep parameters using an accelerometer without sleep diary. Sci Rep.

[45] Padmapriya N, Chen B, Goh CMJL, Shek LPC, Chong YS, Tan KH (2021). 24-hour movement behaviour profiles and their transition in children aged 5.5 and 8 years – findings from a prospective cohort study. Int J Behav Nutr Phys Act.

[46] Hildebrand M, Van Hees VT, Hansen BH, Ekelund ULF (2014). Age group comparability of raw accelerometer output from wrist- and hip-worn monitors. Med Sci Sports Exerc.

[47] Hildebrand M, Hansen BH, van Hees VT, Ekelund U (2017). Evaluation of raw acceleration sedentary thresholds in children and adults. Scand J Med Sci Sports.

[48] Tremblay MS, Aubert S, Barnes JD, Saunders TJ, Carson V, Latimer-Cheung AE (2017). Sedentary behavior research network (SBRN) – terminology consensus project process and outcome. Int J Behav Nutr Phys Act.

[49] Suorsa K, Pulakka A, Leskinen T, Pentti J, Holtermann A, Heinonen OJ (2020). Comparison of Sedentary Time Between Thigh-Worn and Wrist-Worn Accelerometers. J Meas Phys Behav..

[50] Fairclough SJ, Dumuid D, Taylor S, Curry W, McGrane B, Stratton G (2017). Fitness, fatness and the reallocation of time between children’s daily movement behaviours: an analysis of compositional data. Int J Behav Nutr Phys Act.

[51] Dumuid D, Pedišić Ž, Stanford TE, Martín-Fernández JA, Hron K, Maher CA (2019). The compositional isotemporal substitution model: a method for estimating changes in a health outcome for reallocation of time between sleep, physical activity and sedentary behaviour. Stat Methods Med Res.

[52] Chastin SF, Palarea-Albaladejo J, Dontje ML, Skelton DA (2015). Combined effects of time spent in physical activity, sedentary behaviors and sleep on obesity and cardio-metabolic health markers: a novel compositional data analysis approach. PLoS One.

[53] van den Boogaart KG, Tolosana-Delgado R, Bren M. “compositions”: R package to analyze compositional data. 2022. https://cran.r-project.org/web/packages/compositions/index.html . Accessed June 2023.

[54] Dumuid D, Stanford TE, Martin-Fernández J-A, Pedišić Ž, Maher CA, Lewis LK (2017). Compositional data analysis for physical activity, sedentary time and sleep research. Stat Methods Med Res.

[55] Rhodes RE, Guerrero MD, Vanderloo LM, Barbeau K, Birken CS, Chaput J-P (2020). Development of a consensus statement on the role of the family in the physical activity, sedentary, and sleep behaviours of children and youth. Int J Behav Nutr Phys Act.

[56] WHO. WHO guidelines on physical activity and sedentary behaviour. In: World Health Organization, editor. Guideline: https://www.who.int/publications/i/item/9789240015128; 2020. Accessed June 2023.

[57] Chen B, Bernard JY, Padmapriya N, Yao J, Goh C, Tan KH (2019). Socio-demographic and maternal predictors of adherence to 24-hour movement guidelines in Singaporean children. Int J Behav Nutr Phys Act.

[58] Segura-Jiménez V, Pedišić Ž, Gába A, Dumuid D, Olds T, Štefelová N (2023). Longitudinal reallocations of time between 24-h movement behaviours and their associations with inflammation in children and adolescents: the UP&DOWN study. Int J Behav Nutr Phys Act.

[59] Agbaje AO (2023). Longitudinal mediating effect of fat mass and lipids on sedentary time, light PA, and MVPA with inflammation in youth. J Clin Endocrinol Metab..

[60] Agbaje AO, Perng W, Tuomainen T-P (2023). Effects of accelerometer-based sedentary time and physical activity on DEXA-measured fat mass in 6059 children. Nat Commun.

[61] Agbaje AO. Associations of Sedentary Time and Physical Activity From Childhood With Lipids: A 13-Year Mediation and Temporal Study. J Clin Endocrinol Metab. 2023:dgad688. 10.1210/clinem/dgad688. Epub ahead of print. 10.1210/clinem/dgad688 PMC11180508 38097375

[62] Hale L, Emanuele E, James S (2015). Recent updates in the social and environmental determinants of sleep health. Curr Sleep Med Rep.

[63] Downing KL, Hinkley T, Hesketh KD (2015). Associations of parental rules and socioeconomic position with preschool Children's sedentary behaviour and screen time. J Phys Act Health.

[64] Luo W, Zhang Y (2018). Parental expectation and pressure, achievement motivation, and engagement of Singapore students: a self-determination theory perspective. Asian Education Miracles.

[65] Aubert S, Barnes JD, Demchenko I, Hawthorne M, Abdeta C, Abi Nader P (2022). Global matrix 4.0 physical activity report card grades for children and adolescents: results and analyses from 57 countries. J Phys Act Health.

[66] Tay Z, Chen B, Kui KY, Padmapriya N, Chong MF-F, Müller AM (2023). Results from the Singapore 2022 report card on physical activity for children and adolescents. J Exerc Sci Fit..

[67] Petrunoff NA, Edney S, Yi NX, Dickens BL, Joel KR, Xin WN (2022). Associations of park features with park use and park-based physical activity in an urban environment in Asia: a cross-sectional study. Health Place..

