# Supplementary material for: The cross-sectional and prospective associations of parental practices and environmental factors with 24-hour movement behaviours among school-aged Asian children
Source: Int J Behav Nutr Phys Act. 2024 Mar 4;21:27. doi: 10.1186/s12966-024-01574-x (PMC10913559; doi:10.1186/s12966-024-01574-x)
Supplement: Supplementary file 1 — Supplementary Material 1. [file 12966_2024_1574_MOESM1_ESM.docx]

**Supplementary Material 1:** Parental practices and environmental factors

At age 5.5 years, we collected data on parental practices and environmental factors related to movement behaviours of children using a self-administered questionnaire in the Growing Up in Singapore Towards healthy Outcomes (GUSTO) study. The parents were asked to report information on the following items related to parental involvement: (i) How often do parents encourage their child to play outdoors, (ii) how often do the parents physically active with their child or in front of their child, (iii) how often do the parents limit their child’s physical activities as they worry that he/she may injure himself/herself, (iv) how often do the parents focus on developing their child’s basic learning skills, such as numbers and letters, and (v) how often do work/other commitments of the parents limit their time playing with their child. For each item, parents were asked to choose one of the following answers: (a) never, (b) rarely, (c) occasionally, (d) frequently, or (e) all the time. The scores were assigned from 0 to 4 for the first two items and in reverse order (from 4 to 0) for the remaining items.

Parents were also asked to report on how frequently their children were taken to use environmental facilities that encourage physical activity, including (i) open areas, (ii) parks, (iii) playgrounds, (iv) swimming pools, (v) gyms for children, and (vi) clubs for sports/physical activity. For each facility, parents were asked to choose one of the following answers: (a) never, (b) rarely, (c) once a month, (d) a few times a month, (e) once a week, or (f) daily. The scores were assigned from 0 to 5 for all items. Parents were asked if they enrolled their children to participate in extracurricular organised sports/physical activity at least once a week. In addition, parents were asked if their child ate (i) meals or (ii) snacks in front of the television, with the following response options: (a) never, (b) rarely, (c) once a day, (d) twice a day, or (e) 3 or more times a day. The scores were assigned in reverse order (from 4 to 0). Parents were also asked about the presence of a television in their child’s bedrooms.

Parents reported on the availability of facilities for PA in their local neighbourhood, such as parks, playgrounds, and other open areas for PA, as well as facilities for organised sports and physical activity, such as swimming pools, gyms, and sports clubs. The scores ranged from 0 to 3 based on number of facilites available. Parents completed a four-point Likert scale consisting of eight items, with four items related to facilitators of active mobility, including (i) it was safe for their child to play outdoors in their neighbourhood if supervised, (ii) there were usable footpaths on most of the streets in their local area, (iii) there were sufficient traffic lights or pedestrian crossings to make it safe to walk with their child around their neighbourhood, and (iv) the local shop (s) were within easy walking distance of their home. The other four items were related to barriers to active mobility, including (i) there were major barriers or dangers to walking with their child in their neighbourhood that made it hard to get from place to place, (ii) there was so much traffic along the streets that it made it difficult or dangerous to walk with their child in their neighbourhood, (iii) the level of crime in their neighbourhood made it unsafe to go on walks with their child during the day, and (iv) there were dangers (e.g. dogs, undesirable people) in the local park(s) so they avoided taking their child there. The Likert scale ranged from “strongly disagree” (score of 1) to “strongly agree” (score of 4).

Cronbach’s alpha for internal consistency of parental practices variables, environmental factor variables and all variables in parental practices and environmental factors were 0.82, 0.91 and 0.93, respectively. We used principle component analysis (PCA) to explore the underlying dimensions of parental practices measures and environmental factors separately, followed by confirmatory factor analysis (CFA) to confirm the factor structure and generate latent variables. R-package lavaan version 0.6-15 was used to perform CFA (1). Diagonally weighted least squares (DWLS) method was used to estimate factor loadings, as our observed variables are ordinal data (2).

We identified three factors for parental practices and three factors for environmental factors, which were in line with the items grouped in the questionnaire and conceptually meaningful, namely “parental involvement in PA”, “parental support (on using environmental facilities) for PA”, “parental control on screen viewing context”, “facilities for active play or PA”, “facilitators for active mobility” and “barriers to active mobility”. However, factor loading for the following items was very low (<0.1) and were removed from the construct: (i) how often do work/other commitments of the parents limit their time playing with their child and (ii) how often do the parents focus on developing their child’s basic learning skills. The final model demonstrated a good fit based on multiple indices [Comparative Fit Index (CFI) = 0.937; Tucker-Lewis Index (TLI) = 0.925; Root Mean Square Error of Approximation (RMSEA) = 0.065; Standardized Root Mean Square Residual (SRMR) = 0.076). The model’s Chi-square value was significant (p < 0.001)]. Discriminant validity was examined by comparing the correlations between each pair of constructs. This comparison was conducted using a chi-square test, the results of which revealed significant differences between the constructs of the factors (p < 0.05), thus confirming the discriminant validity of the factors in the model. The factor loadings of final model is illustrated in Supplementary Figure 1.

Supplementary Figure 1: Parental practices and environmental factors at age 5.5 years in the GUSTO study

*statistically significant (p<0.001)

Reference

1. Rosseel Y. lavaan: An R Package for Structural Equation Modeling. Journal of Statistical Software. 2012;48(2):1 - 36.

2. Li C-H. Confirmatory factor analysis with ordinal data: Comparing robust maximum likelihood and diagonally weighted least squares. Behavior Research Methods. 2016;48(3):936-49.
