# Supplementary material for: The cross-sectional and prospective associations of parental practices and environmental factors with 24-hour movement behaviours among school-aged Asian children
Source: Int J Behav Nutr Phys Act. 2024 Mar 4;21:27. doi: 10.1186/s12966-024-01574-x (PMC10913559; doi:10.1186/s12966-024-01574-x)
Supplement: Supplementary file 2 — Supplementary Material 2. [file 12966_2024_1574_MOESM2_ESM.docx]

Supplementary Table 1: Cross-sectional and prospective associations of parental practices with accelerometer-measured 24-hour movement behaviours in the GUSTO cohort

|  | **Unadjusted model** | | | | | **Adjusted model^a^** | | | | |
| --- | --- | --- | --- | --- | --- | --- | --- | --- | --- | --- |
|  | **Relative to remaining behaviours** | | | | **Overall**  **p-value*** | **Relative to remaining behaviours** | | | | **Overall p-value*** |
|  | **MVPA** | **LPA** | **Inactivity** | **Sleep** |  | **MVPA** | **LPA** | **Inactivity** | **Sleep** |  |
|  | Mean difference (95% CI) | Mean difference (95% CI) | Mean difference (95% CI) | Mean difference (95% CI) |  | Mean difference (95% CI) | Mean difference (95% CI) | Mean difference (95% CI) | Mean difference (95% CI) |  |
| **24-hour movement behaviours at age 5.5 years (n=544)** | | | | | | | | | | |
| Parental involvement | **0.043**  **(0.016, 0.071)** | 0.010  (-0.002, 0.022) | **-0.043**  **(-0.063, -0.024)** | -0.010  (-0.022, 0.002) | **<0.001** | **0.043**  **(0.015, 0.072)** | 0.008  (-0.004, 0.020) | **-0.040**  **(-0.061, -0.019)** | -0.011  (-0.024, 0.001) | **0.003** |
| Parental support for PA | **0.043**  **(0.015, 0.071)** | 0.009  (-0.003, 0.021) | **-0.044**  **(-0.063, -0.024)** | -0.009  (-0.021, 0.004) | **<0.001** | **0.046**  **(0.018, 0.075)** | 0.005  (-0.007, 0.018) | **-0.041**  **(-0.062, -0.020)** | -0.011  (-0.024, 0.002) | **0.002** |
| Parental control on screen viewing context | -0.004  (-0.030, 0.023) | 0.008  (-0.003, 0.019) | -0.015  (-0.035, 0.004) | 0.011  (-0.001, 0.023) | 0.009 | 0.003  (-0.026, 0.031) | 0.001  (-0.011, 0.013) | -0.014  (-0.034, 0.007) | 0.010  (-0.003, 0.023) | 0.054 |
| Overall parental practices | 0.034  (0.007, 0.062) | 0.012  (0.000, 0.023) | **-0.043**  **(-0.063, -0.024)** | -0.003  (-0.015, 0.010) | **<0.001** | **0.039**  **(0.010, 0.068)** | 0.006  (-0.006, 0.019) | **-0.040**  **(-0.061, -0.020)** | -0.005  (-0.018, 0.008) | **0.001** |
| **24-hour movement behaviours at age 8 years (n=568)** | | | | | | | | | | |
| Parental involvement | **0.059**  **(0.030, 0.088)** | 0.012  (-0.001, 0.026) | **-0.046**  **(-0.066, -0.027)** | **-0.025**  **(-0.038, -0.012)** | **<0.001** | **0.057**  **(0.028, 0.085)** | 0.004  (-0.010, 0.018) | **-0.035**  **(-0.055, -0.015)** | **-0.025**  **(-0.039, -0.012)** | **<0.001** |
| Parental support for PA | **0.042**  **(0.014, 0.070)** | **0.015**  **(0.002, 0.028)** | **-0.038**  **(-0.058, -0.019)** | **-0.019**  **(-0.032, -0.006)** | **0.001** | **0.039**  **(0.011, 0.067)** | 0.007  (-0.007, 0.020) | **-0.027**  **(-0.047, -0.007)** | **-0.019**  **(-0.032, -0.005)** | **0.023** |
| Parental control on screen viewing context | -0.003  (-0.032, 0.026) | **0.011**  **(-0.002, 0.024)** | -0.011  (-0.031, 0.009) | 0.003  (-0.011, 0.016) | 0.242 | 0.003  (-0.026, 0.032) | 0.006  (-0.008, 0.019) | -0.006  (-0.026, 0.015) | -0.002  (-0.016, 0.011) | 0.865 |
| Overall parental practices | **0.042**  **(0.013, 0.071)** | 0.017  (0.004, 0.030) | **-0.041**  **(-0.061, -0.022)** | **-0.018**  **(-0.031, -0.004)** | **<0.001** | **0.043**  **(0.014, 0.072)** | 0.007  (-0.007, 0.021) | **-0.030**  **(-0.051, -0.009)** | **-0.020**  **(-0.034, -0.007)** | **0.012** |
| MD, mean difference; CI, confidence interval; MB, movement behaviour; SB, sedentary behaviour; LPA, Light physical activity; MVPA, Moderate-to-vigorous physical activity  ^a^Models were adjusted for sex, ethnicity, BMI at age 5.5 years and maternal age and education;  Results are based on a compositional data analysis, multivariate linear regression models;  *Type II MANOVA Tests: Pillai test statistics | | | | | | | | | | |
