# Supplementary material for: The cross-sectional and prospective associations of parental practices and environmental factors with 24-hour movement behaviours among school-aged Asian children
Source: Int J Behav Nutr Phys Act. 2024 Mar 4;21:27. doi: 10.1186/s12966-024-01574-x (PMC10913559; doi:10.1186/s12966-024-01574-x)
Supplement: Supplementary file 3 — Supplementary Material 3. [file 12966_2024_1574_MOESM3_ESM.docx]

Supplementary Table 2: Cross-sectional and prospective associations of environmental factors with accelerometer-measured 24-hour movement behaviours in the GUSTO cohort

|  | **Unadjusted model** | | | | | **Adjusted model^a^** | | | | |
| --- | --- | --- | --- | --- | --- | --- | --- | --- | --- | --- |
|  | Relative to remaining behaviours | | | | **Overall**  **p-value*** | Relative to remaining behaviours | | | | **Overall**  **p-value*** |
|  | **MVPA** | **LPA** | **Inactivity (SB)** | **Sleep** |  | **MVPA** | **LPA** | **Inactivity (SB)** | **Sleep** |  |
|  | Mean difference (95% CI) | Mean difference (95% CI) | Mean difference (95% CI) | Mean difference (95% CI) |  | Mean difference (95% CI) | Mean difference (95% CI) | Mean difference (95% CI) | Mean difference (95% CI) |  |
| **24-hour movement behaviours at age 5.5 years (n=544)** | | | | | | | | | | |
| Facilities for active play | 0.027  (0.000, 0.055) | 0.010  (-0.002, 0.021) | **-0.033**  **(-0.053, -0.014)** | -0.004  (-0.016, 0.008) | **0.006** | **0.031**  **(0.003, 0.059)** | 0.005  (-0.007, 0.017) | -0.031  (-0.051, 0.010) | -0.006  (-0.019, 0.007) | **0.033** |
| Facilitators for active mobility | 0.018  (-0.010, 0.045) | -0.002  (-0.014, 0.010) | -0.016  (-0.036, 0.004) | 0.000  (-0.012, 0.012) | 0.087 | 0.017  (-0.011, 0.044) | -0.004  (-0.016, 0.008) | -0.013  (-0.033, 0.007) | 0.000  (-0.012, 0.012) | 0.209 |
| Barriers to active mobility | -0.005  (-0.021, 0.032) | 0.001  (-0.011, 0.012) | -0.014  (-0.033, 0.005) | 0.008  (-0.004, 0.019) | 0.272 | 0.004  (-0.023, 0.031) | -0.003  (-0.015, 0.008) | -0.008  (-0.028, 0.011) | 0.007  (-0.005, 0.020) | 0.377 |
| Overall environmental factors | 0.020  (-0.007, 0.047) | 0.003  (-0.009, 0.015) | -0.026  (-0.045, 0.006) | 0.002  (-0.010, 0.014) | **0.022** | 0.021  (-0.007, 0.049) | -0.001  (-0.013, 0.011) | -0.021  (-0.042, 0.001) | 0.001  (-0.011, 0.014) | 0.089 |
| **24-hour movement behaviours at age 8 years (n=568)** | | | | | | | | | | |
| Facilities for active play | 0.028  (0.000, 0.056) | **0.018**  **(0.006, 0.031)** | **-0.034**  **(-0.053, -0.014)** | -0.013  (-0.026, 0.000) | **0.002** | 0.023  (-0.004, 0.051) | 0.012  (-0.001, 0.025) | **-0.023**  **(-0.043, -0.004)** | -0.012  (-0.025, 0.002) | 0.072 |
| Facilitators for active mobility | **0.034**  **(0.005, 0.064)** | 0.005  (-0.008, 0.019) | **-0.026**  **(-0.047, -0.006)** | -0.013  (-0.027, 0.000) | 0.201 | 0.024  (-0.004, 0.052) | 0.003  (-0.011, 0.016) | -0.017  (-0.037, 0.003) | -0.010  (-0.023, 0.004) | 0.580 |
| Barriers to active mobility | 0.027  (-0.002, 0.055) | 0.000  (-0.013, 0.014) | **-0.021**  **(-0.041, -0.001)** | -0.006  (-0.019, 0.007) | 0.076 | 0.018  (-0.009, 0.046) | -0.003  (-0.016, 0.010) | -0.010  (-0.030, 0.009) | -0.004  (-0.017, 0.009) | 0.343 |
| Overall environmental factors | **0.039**  **(0.010, 0.068)** | 0.010  (-0.003, 0.023) | **-0.035**  **(-0.055, -0.015)** | -0.014  (-0.028, 0.000) | **0.008** | 0.029  (-0.001, 0.055) | 0.004  (-0.009, 0.018) | **-0.022**  **(-0.043, -0.002)** | -0.011  (-0.025, 0.002) | 0.167 |
| CI, confidence interval; SB, sedentary behaviour; LPA, Light physical activity; MVPA, Moderate-to-vigorous physical activity  ^a^Models were adjusted for sex, ethnicity, BMI at age 5.5 years and maternal age and education  Results are based on a compositional data analysis, multivariate linear regression models;  *Type II MANOVA Tests: Pillai test statistics | | | | | | | | | | |
